# Validity of Diagnostic Support Model for Attention Deficit Hyperactivity Disorder: A Machine Learning Approach

**DOI:** 10.3390/jpm13111525

**Published:** 2023-10-24

**Authors:** Kuo-Chung Chu, Hsin-Jou Huang, Yu-Shu Huang

**Affiliations:** 1 Department of Information Management, National Taipei University of Nursing and Health Sciences, Taipei 112, Taiwan; kcchu@ntunhs.edu.tw (K.-C.C.);; 2 Department of Education and Research, Taipei City Hospital, Taipei 103, Taiwan; 3 Department of Child Psychiatry and Sleep Center, Chang Gung Memorial Hospital at Linkou, Taoyuan City 333, Taiwan; 4 College of Medicine, Chang Gung University, Taoyuan 333, Taiwan

**Keywords:** attention deficit hyperactivity disorder, clinical diagnosis support, receiver operating characteristic curve, machine learning

## Abstract

An accurate and early diagnosis of attention deficit hyperactivity disorder can improve health outcomes and prevent unnecessary medical expenses. This study developed a diagnostic support model using a machine learning approach to effectively screen individuals for attention deficit hyperactivity disorder. Three models were developed: a logistic regression model, a classification and regression tree (CART), and a neural network. The models were assessed by using a receiver operating characteristic analysis. In total, 74 participants were enrolled into the disorder group, while 21 participants were enrolled in the control group. The sensitivity and specificity of each model, indicating the rate of true positive and true negative results, respectively, were assessed. The CART model demonstrated a superior performance compared to the other two models, with region values of receiver operating characteristic analyses in the following order: CART (0.848) > logistic regression model (0.826) > neural network (0.67). The sensitivity and specificity of the CART model were 78.8% and 50%, respectively. This model can be applied to other neuroscience research fields, including the diagnoses of autism spectrum disorder, Tourette syndrome, and dementia. This will enhance the effect and practical value of our research.

## 1. Introduction

Attention deficit hyperactivity disorder (ADHD) is the most common neurodevelopmental disorder among children and teenagers. Its symptoms in youths include inattentiveness, poor impulse and emotional control, and hyperactivity. Youths with ADHD tend to be disruptive in classroom environments and have learning and socialization difficulties and mood disorders [1]. Childhood ADHD and conduct problems are associated with adult criminal activity. Impulsivity is associated with the likelihood of injury due to accidents, violent behavior, and suicidal ideation. One definition of impulsivity is the tendency to act without exercising self-control. An individual who can comprehend the consequences of their actions may still be considered impulsive [2].

The global prevalence of ADHD among school-aged individuals has seen an increase [3,4]. The Diagnostic and Statistical Manual of Mental Disorders [5] identifies three primary presentations of ADHD: predominantly inattentive, predominantly hyperactivity–impulsivity, and a combination of the two. In a study conducted by Willcutt [6], which examined 86 studies published between 1994 and 2010, the prevalence rates of ADHD among accurately diagnosed children were found to be 1.8% for the predominantly inattentive presentation, 0.8% for the predominantly hyperactivity–impulsivity presentation, and 3.4% for the combination of both presentations. According to the Diagnostic and Statistical Manual of Mental Disorders, Fifth Edition (DSM-5) published in 2013 [7], the age at which symptoms typically appear has been revised to encompass children between the ages of 7 and 12. Additionally, teenagers aged 17 and above, as well as adults exhibiting five symptoms, can also meet the diagnostic criteria. In practice, ADHD is diagnosed by evaluating a child’s behavioral and cognitive progress, often incorporating input from parents and teachers through questionnaires. For example, Yasin et al. [8] examined the relationship between pathological internet use, aggression, and cyberbullying/victimization in children with ADHD [8], whereas Yılbaş et al. [9] investigated the connection between chronotypes, impulsivity, attention-deficit disorder, and smartphone, social media, and internet addiction in a sample of university students. ADHD symptoms vary by age and sex, rendering its diagnosis challenging. A study that used electroencephalography observed significant gender differences in ADHD symptoms [10,11]. With advancements in medical technology and treatment techniques and an increase in the amount of clinical data, the value of machine learning has been increasingly recognized, leading to its application in making diagnoses, predicting diseases, and enabling the more efficient processing of large amounts of data [12].

Recent studies have investigated the use of machine learning technologies in an ADHD diagnosis. For example, association rule mining was applied to analyze ADHD-related comorbidity by using data from the National Health Insurance Research Database in Taiwan [13]. To evaluate the mediating role of anxiety and depressive symptoms in the relationship between ADHD and academic performance, a logit model and mediation analyses were carried out to test whether the relationship between ADHD symptoms and academic performance might be mediated by depressive and anxiety symptoms [14]. This study employed a logistic regression model, a classification and regression tree (CART), and a neural network machine learning algorithm to develop predictive models for ADHD. To evaluate and compare the accuracy of these models, a receiver operating characteristic (ROC) analysis was conducted.

## 2. Materials and Methods

### 2.1. Data Collection

The participant selection process relied on evaluations performed by a team of experienced child psychologists at a medical center. The participants underwent the test of variables of attention (TOVA) [15,16], which involves following rules and responding to stimuli of target objects and distractors (non-target objects), so that response behavior and attention expression can be analyzed. The time taken to respond to stimuli is evaluated and used to measure impulsivity. When a target object appears, the participant must respond, and the number of times the participant fails to respond is recorded. When a distractor appears, the participant should ignore it, and the number of times the participant does not ignore it is recorded. The six variables measured were (1) omission errors: the number of targets that were missed, (2) commission errors: the number of responses to distractors, (3) response time: mean response latency in milliseconds, (4) variability: the standard deviation of response times, (5) D Prime: the concentration degree and sensitivity, calculated based on the omission error and commission error percentages, and (6) ADHD score: a comprehensive diagnostic index that encompasses response time, variability, and D Prime. 

Inattention was evaluated by dividing the number of times an omission error was made by the total number of target objects (with the subtraction of the number of anticipatory responses that were made). Impulsivity was evaluated by dividing the number of commission errors that were made by the total number of distractors (with the subtraction of the number of anticipatory responses that were made). 

The test included six stages (Table 1). Each stage had 125 objects (target objects and distractors). The ratios of target objects to distractors were 1:4 in the first half of the test (S1–S3) and 4:1 in the second half of the test (S4–S6). Objects were presented at a rate of 30 per min. The test took 25 min to complete. Scores were compared with those of a group of the same age and are reported as standard deviations. Normal scores were between −1.00 and 1.00. The lower the standard deviation, the greater the problem.

### 2.2. Establishing a Valid ADHD Diagnostic Support Model

Several studies have investigated the application of machine learning in medicine [17,18,19,20]. Machine learning can be used to improve the accuracy of clinical diagnoses, which affects subsequent medication prescription. Patient assessments and clinical diagnoses involve comprehensive analyses by using various instruments (including tests and questionnaires). Physicians have limited time to provide diagnoses; therefore, an auxiliary decision-making system is crucial because it can expedite case screening, prevent unnecessary expenses due to repeat diagnoses, and minimize risks associated with misdiagnoses.

**Figure 1:**
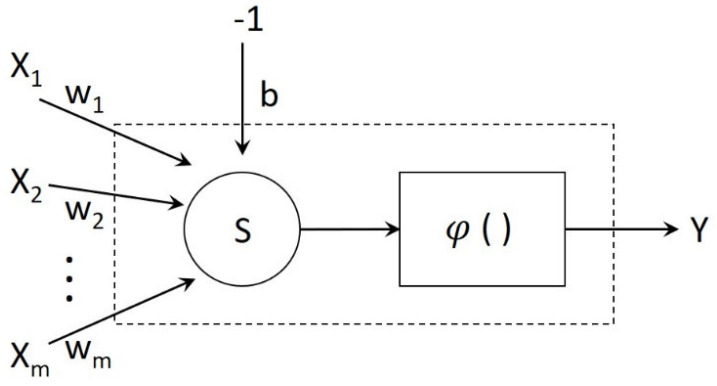
Neural network diagnosis model.

To achieve an adequate performance and improve the accuracy of an ADHD prediction and diagnosis, diagnostic support models rely on valid indices. We developed the following three diagnostic support models:

1. Logistic regression model. In this model, *X* is the set of the diagnosis index, *Y* is the set of all cases, *x_ij_* is the [*j* (*j*∈*Y*)]th index of the test of case *i* (*i*∈, *X*), and *y_i_* is a binary variable that indicates whether case *i* involves ADHD (1 indicating YES and 0 indicating NO). The regression model function can thus be expressed as *y_i_* = F(*x_i1_, x_i2_, ………, x_ij_*).
2. CART [21,22,23,24]. This process involves applying a single input variable to separate the data in each node, thereby constructing a binary decision tree in which the variable for each decision node is automatically determined by the CART algorithm. The selection of the appropriate variable depends on the node splitting method, which may be the Gini index, entropy, or information gain [21,22]. By ensuring that each node has only one input variable, we can compare the value of the sample variable with a decision rule to obtain a prediction outcome. The first task is to determine which independent variable is the optimal separation variable. The optimal separation variable can categorize data into a singular group by using splitting methods. By using the optimal separation variable, diversity within a data set can be minimized. Data-set diversity can be evaluated by using the following formula: diversity (before separation) − (diversity (left subset after separation) + diversity (right subset after separation)).
3. Neural network [25,26]. The neural network is shown in Figure 1. *X_i_* is the neuron input, which is the index that predicts ADHD; *W_i_* is the weight of *X_i_*; b is the bias that can be used as a limited value to enhance the model; *S* is the summation, in which each input is multiplied by the weight and then totaled; *φ*( ) is the activation function, which is usually a nonlinear function, includes various types, and is the output required to map the value of *S*; and *Y* is the output, which indicates whether an individual has ADHD.

**Figure 2:**
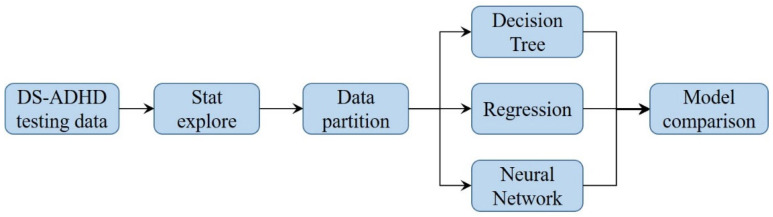
Diagnosis aid model.

The ADHD score is the output (dependent) variable, and the other five variables are the input (independent) variables. The three models were trained and tested in SAS Enterprise Miner (EM) 14.2, Cary, North Carolina, USA [27], and the results were analyzed to determine sensitivity and specificity. The models were compared. The diagnosis aid model is shown in Figure 2. For the neural network, the layer(s), neuron(s) in each layer, and activation function were set to the default parameters of SAS EM. The models were trained and tested using 70% of the samples and 30% of the samples in the data set, respectively. The training and the testing data sets included the data of 67 and 28 participants, respectively.

### 2.3. Diagnostic Efficacy Analysis

We utilized an ROC analysis to evaluate and compare the effectiveness of the diagnostic support models. The discriminative ability of the models was assessed using a 2 × 2 confusion matrix, as represented in Table 2. For a comprehensive understanding of an ROC analysis, please refer to [28] for detailed information supporting its application.

Sensitivity and specificity make up the effectiveness index of the ROC analysis and represent the ratio of true positive (TP) and true negative (TN), respectively.

(1) $$\mathrm{Sensitivity}=\frac{TP}{TP+FN}$$

(2) $$\mathrm{Specificity}=\frac{TN}{TN+FP}$$

## 3. Results

We enrolled participants with ADHD who had been diagnosed according to the DSM-5 criteria. Subsequently, we conducted an analysis using data from a sample of 95 children, aged 6 to 12 years. Among them, there were 74 children in the ADHD group and 21 in the non-ADHD (control) group, with an average age of 9.18, as shown in Table 3.

**Figure 3:**
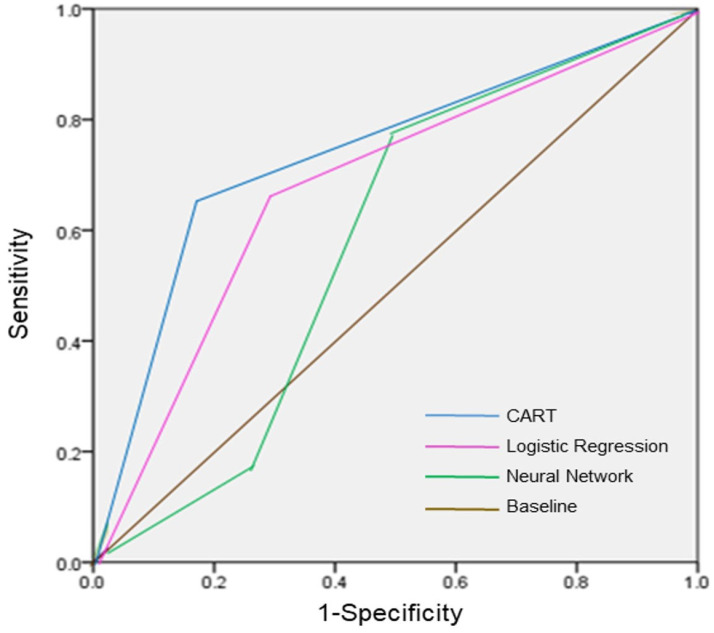
ROC curve for three ADHD diagnostic support models.

The results of the ROC analysis are shown in Figure 3. The CART was the best of the three models; the ROC region values are listed in the following order: CART (0.848) > logistic regression model (0.826) > (0.67). We additionally assessed the testing data set in the three models; with respect to a specificity of 50%, the sensitivity of the CART, logistic regression, and neural network were 78.8%, 78.5%, and 78.7%, respectively.

The CART was more effective than the logistic regression model and neural network, with the response time being a critical decision variable.

**Figure 4:**
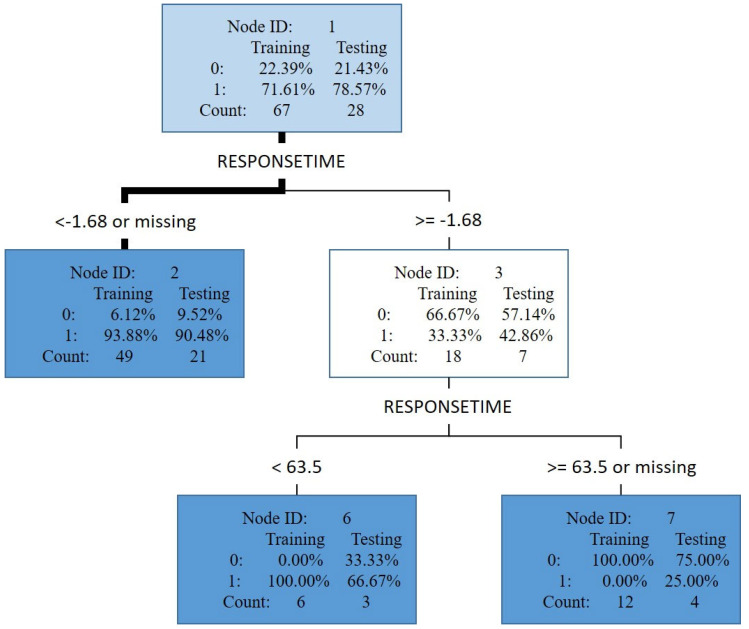
CART diagnosis prediction result.

Figure 4 displays the result of the trimmed tree, where rectangular tree node ID 2 represents a subset of the testing data set with a response time less than −1.68 milliseconds. This testing data set had a count of 21. The likelihood of having ADHD was 90.48% (count = 19), as indicated by the value of 1 in the node, and the likelihood of not having ADHD was 9.52% (count = 2), as indicated by the value of 0 in the node. Furthermore, when the response time was more than 63.5 (in node ID 7), the likelihood of not having ADHD (75%) was higher than that of having ADHD (25%).

## 4. Discussion

Based on our analysis, the CART model emerged as the most effective among the three diagnostic aid models. We observed that participants with ADHD exhibited longer response times compared to those without ADHD, possibly due to factors such as impatience and inattention. Children with ADHD also displayed a higher likelihood of repeatedly and incorrectly responding to target objects. Additionally, children with ADHD had slower response times compared to those without ADHD [29].

While the three models performed similarly, the CART model demonstrated a higher sensitivity than the other two models. Sensitivity, which is more crucial in distinguishing between ADHD and non-ADHD cases, was consistently higher than specificity across all three models [30]. This distinction holds greater importance as delayed treatment can significantly impact an individual’s overall functioning and development. The specificity, however, was only 50%, likely influenced by the imbalanced data set and sample size (with an unequal number of participants with and without ADHD). Achieving a specificity of 50% makes it challenging to accurately distinguish individuals with ADHD. Nevertheless, screening out ADHD cases is more critical than identifying non-ADHD cases, so we focused on the model with a higher sensitivity.

It is important to discuss the implications of the disparity in the number of ADHD and non-ADHD cases. Firstly, the substantial difference in sample sizes between the two groups may impact the statistical power and generalizability of the study findings. The larger number of ADHD participants introduces the potential for bias and limitations when drawing conclusions about the characteristics and outcomes of both groups. Addressing this imbalance and acknowledging its effect on the study’s validity is crucial. Secondly, the uneven distribution of cases and controls may introduce confounding factors and limitations when comparing the two groups. Factors like comorbid conditions, treatment history, and demographic variables could vary between the groups, influencing the interpretation of results. Recognizing and addressing these potential confounders is essential for a comprehensive analysis and accurate interpretation of the findings.

Furthermore, the disproportionate ratio raises concerns about the representativeness of the study sample and its generalizability to the broader population. Understanding the potential biases introduced by the sample composition is crucial for interpreting the study’s implications and considering its applicability in real-world scenarios.

By discussing the implications of the imbalance between cases and controls, we acknowledge potential limitations, address biases, and enhance the interpretation and generalizability of the study findings within the existing literature on ADHD.

## 5. Conclusions

This study aimed to propose and evaluate the validity of a machine learning model for the clinical screening of ADHD in children. Three models, including a logistic regression model, a CART, and a neural network, were developed. The CART model exhibited a superior performance in predicting ADHD status compared to the other two models. The study’s primary goal was to enhance the efficiency of ADHD screening, reducing unnecessary medical expenses associated with repeat diagnoses. Additionally, the use of the CART model minimizes the risk of a misdiagnosis, improves the objective assessment of drug efficacy, and holds potential for application in other neuroscience fields.

Children with ADHD often experience comorbidities that complicate an accurate diagnosis and increase the likelihood of errors or incorrect assessments. This may explain the lower specificity observed in the ROC analysis results. Future research should consider incorporating the influence of comorbidities. Furthermore, due to the limited sample size, the study did not investigate the impact of gender. Future studies with sample sizes larger than 100 are warranted to explore the potential influence of gender. Additionally, it would be valuable to investigate the relationship between ADHD and substance use, as well as the effects of psychopharmacological therapy.

Regarding substance use, future research can delve into the potential link between ADHD and substance use, examining how substance use affects ADHD symptoms and vice versa. Understanding the prevalence of substance use among individuals with ADHD, the types of substances commonly used, and the impact of substance use on symptom severity, treatment outcomes, and overall functioning would be beneficial.

In terms of psychopharmacological therapy, it is essential to investigate the effects of different medications on ADHD symptoms. This includes examining their efficacy, potential side effects, optimal dosages, and long-term outcomes. Research efforts could also focus on identifying biomarkers or predictors of the treatment response to guide personalized medication selection and improve treatment effectiveness.

By studying these variables, researchers can gain valuable insights into the complex interplay between ADHD, substance use, and psychopharmacological therapy. This knowledge can inform diagnostic practices, treatment approaches, and interventions aimed at better supporting individuals with ADHD and improving their overall well-being.

## Figures and Tables

**Table 1:** Participant Characteristics.

	Number	Age
ADHD group	74	9.32
Non-ADHD group	21	8.70
Overall	95	9.18

**Table 2:** Numbers of Target Objects and Distractors in Each Stage of Test of Variables of Attention.

	Stage						Half		Total
	S1	S2	S3	S4	S5	S6	H1	H2	
Target object	25	25	25	100	100	100	75	300	375
Distractor	100	100	100	25	25	25	300	75	375

**Table 3:** Model Performance Evaluation Results by Using ROC Analysis.

	Has ADHD	Does Not Have ADHD
Predicted to have ADHD	True Positive	False Negative
Predicted to not have ADHD	False Positive	True Negative

## Data Availability

The data that support the findings of this study are available on reasonable request from the corresponding author.
